# Exercise-Based Cardiac Rehabilitation: Secondary Data Analyses of Mortality and Working Capacity in Germany, 2010–2017

**DOI:** 10.1186/s40798-021-00381-z

**Published:** 2021-12-04

**Authors:** Lars Gabrys, Johannes Soff, Christian Thiel, Christian Schmidt, Enno Swart, Dirk Peschke

**Affiliations:** 1 Department of Sport and Prevention, University of Applied Sciences for Sport and Management Potsdam, Am Luftschiffhafen 1, 14471 Potsdam, Germany; 2 Department of Applied Health Sciences, University of Applied Sciences for Health, Bochum, Germany; 3 Department of Epidemiology and Health Monitoring, Robert Koch Institute, Berlin, Germany; 4 Institute for Social Medicine and Health System Research, Otto Von Guericke University Magdeburg, Magdeburg, Germany

**Keywords:** Exercise, Physical activity, Sports, Rehabilitation, Health care, Cardiology, Epidemiology

## Abstract

**Background:**

Exercise-based cardiac rehabilitation is safe and implemented in international cardiac rehabilitation guidelines. Evidence for long-term health effects is scarce and rare for health care service research.

**Objective:**

The aim of this study is to evaluate the effectiveness of exercise-based phase III cardiac rehabilitation programs in improving mortality and working capacity outcomes.

**Methods:**

The present analyses used claims data of the German pension fund from 2010 to 2017. Overall, 54,163 patients with coronary heart disease (ICD10 I20.–I25.) were included and followed up for exercise-based cardiac rehabilitation participation (mean 4.3 ± 1.9 years). All patients were categorized according to participation duration (long: ≥ 90 days, short: < 90 days, no). The effectiveness of exercise-based rehabilitation was analyzed by calculating adjusted hazard ratios for mortality and reduced working capacity in relation to program participation.

**Results:**

Of all the cardiac patients, 57.6% received medical recommendations for exercise-based phase III rehabilitation, and 16.8% participated in this rehabilitation. In total, 1776 (3.3%) patients died during the study period, and 3050 (5.5%) received reduced earning capacity pensions. Mortality risk was nearly doubled for those who did not participate in exercise-based cardiac rehabilitation compared to those who participated for a long duration (HR 1.97, 95% CI 1.60–2.43) and 44% higher compared to a short participation (HR 1.44, 95% CI 1.03–2.01). Furthermore, the risk of reduced working capacity was higher for those who did not participate compared to those who participated for a short duration (HR 1.24, 95% CI 1.00–1.54).

**Conclusion:**

Exercise-based phase III cardiac rehabilitation is independently associated with reduced mortality and reduced loss in working capacity. Strong efforts should be made to increase participation rates to improve cardiac patients care.

## Key Points

- Outpatient exercise-based cardiac rehabilitation (phase III) is a cornerstone to induce long-term health behavior change and stabilize health outcomes. Patients who adhere to these programs show lower mortality rates and a better working capacity compared to cardiac patients who do not participate.
- Although exercise-based cardiac rehabilitation is implemented in national and international guidelines, only 16.8% of cardiac patients are successfully referred to structured rehabilitation programs in Germany.
- Strong efforts should be made to increase participation rates to improve cardiac patients care. Digital tools like cardiac telerehabilitation could make exercise-based cardiac rehabilitation more widely accessible and tailored to individual needs.

## Introduction

Coronary heart disease (CHD) or ischemic heart disease is the second leading cause of disability-adjusted life years (DALYs) worldwide. It is the leading cause of DALYs among persons over 50 years of age [1]. In Germany, CHD exhibits a lifetime prevalence of 6.4% in women and 12.3% in men, and the prevalence of this condition increases with age [2, 3]. In 2019, more than 330,000 deaths in Germany were attributable to cardiovascular diseases [4] and 838,000 hospital admissions due to CHD were documented nationwide [5]. Although CHD incidence rates and cardiovascular mortality have decreased over the last few decades [2], morbidity, reduced physical capacity and physical functioning, and premature death remain high in CHD survivors [6].

The German healthcare system offers inpatient or outpatient rehabilitation care for CHD patients, and the associated costs are covered by social insurance agencies. Depending on the patient’s age and working ability, rehabilitation measures must be paid for by statutory health insurances, the German pension fund (DRV), or private health insurances (e.g., for patients who have higher-income levels, are self-employed, or are civil servants). For the majority of the working population, the German pension fund covers rehabilitation costs.

After a cardiac event or elective cardiac surgery, the rehabilitation process within the German healthcare system comprises three sequential rehabilitation stages. The first stage (Phase I) focuses on intensive care and early mobilization within the hospital. The second stage (Phase II) includes a 3–4-week inpatient or outpatient medical rehabilitation program with a focus on improving physical capacity, disease management, quality of life, and return to work [7]. Exercise and sports therapy are major components of Phase II cardiac rehabilitation [8, 9]. There is clear evidence for the prognostic significance of exercise training and cardiorespiratory fitness, which are inversely related to cardiovascular mortality. Further, the antiatherosclerotic, antiischemic, antiarrhythmic, antithrombotic, and psychological mechanisms of regular physical activity and exercise, responsible for the decreased mortality are well known [10]. Exercise-based cardiac rehabilitation is safe and effective, can reduce cardiovascular mortality by 26% and rehospitalization by 18%, and can also improve both health-related and overall quality of life [9, 11, 12]. To stabilize treatment outcomes and support long-term lifestyle and behavior changes, cardiac patients are encouraged to attend exercise-based outpatient aftercare programs in Phase III rehabilitation (EBRP-III). In these exercise-based group programs, patients should exercise regularly at or near their places of residence to improve health, reduce cardiovascular risk, and prevent the loss of working capacity and early retirement. Typically, EBRP-III is prescribed by a physician (often a general practitioner), should start within three months after Phase II, and should be continued for as long as possible. It involves 1–2 regular group exercise sessions per week, each lasting for at least 60 min and guided by a rehabilitation trainer under the surveillance of a physician. These rehabilitation trainers and programs must be licensed and listed by either the German Society of Cardiovascular Prevention and Rehabilitation (DGPR) or the German National Paralympic Committee (DBS). A prescription within the scope of the German pension fund typically covers a period of six months. Rehabilitation goals, such as body functioning and participation, are in accordance to the International Classification of Functioning (ICF) [13, 14].

In 2014, 5% (N = 46,894) of all Phase II rehabilitation measures of the German pension fund were related to CHD (e.g., myocardial infarction). Of these patients, approximately 60% received prescriptions for EBRP-III [15]. Participation analyses have shown that only a minority (9.7–22.5%) of CHD patients have actually participated in EBRP-III [16]. Furthermore, little is known as to whether these patients benefit from program participation.

The present paper uses claims data to analyze the effects of EBRP-III on CHD patients in terms of mortality and lost working capacity.

## Methods

### Data Basis

We used claims data of the German pension fund for the present analyses. The Scientific Use File (SUF) “completed rehabilitation measures 2010–2017” (Abgeschlossene Rehabilitationen 2010–2017 [in German]) is available upon request for scientific institutions. The dataset contains person-based data with records on insured persons and their eligible relatives. Included are persons with one of the following characteristics within the observed period (2010–2017): completed rehabilitation, approved pension or belonging to a specific demographic cohort (death before or at the age of 75 or belonging to a certain year of birth). Persons whose applications for rehabilitation or for any pension had finally been denied are excluded. For each rehabilitation, pension, or demographic event, included persons are being observed over an 8-year period, with minor exceptions (e.g. date of death information is available up to 9 years after the first documented event.) The SUF is a complex random sample regarding the aforementioned events and comprises a random sample of 20% of all insured persons (N ≈ 3.7 million). We used the given weighting factor to infer from the random sample to the target population. Given that the DRV is the main payer for rehabilitation services for the working population of Germany, the age range of the participants in this dataset is typically between 16 and 66 years. A detailed description of the dataset, including the sampling design, can be found elsewhere [17, 18]. Due to its longitudinal design (with individual data from 2010 to 2017), the SUF is an appropriate source for the analysis of the effects and time trends of rehabilitation services.

The study adheres to the reporting guidelines of the STROBE Statement for observational studies [19].

### Participants and Outcomes

Out of the complete dataset of 3.7 million insured persons, we only used data with information on medical rehabilitation measures for our analyses. For these approximately 2.2 million patients we defined the following inclusion and exclusion criteria.

#### Inclusion Criteria

In the first step, we included rehabilitation patients with a primary diagnosis of CHD or ischemic heart disease (ICD10 I20.–I25.) who had completed medical rehabilitation (Phase II). In the next step, we matched EBRP-III to our study population. Participation had to start within six months after completing Phase II rehabilitation.

#### Exclusion Criteria

People who died within a 12-month period after completing Phase II rehabilitation were excluded to avoid selection bias due to the inclusion of severely ill patients. We also excluded persons without regularly completed rehabilitation phase II because of medical problems or premature termination of any other cause.

The time of study entry was determined by the date of the first use of Phase II rehabilitation services, which could have been any date after January 1, 2010. Patients were followed up for mortality or reduced working capacity as primary study outcomes until December 31, 2017. The loss of working capacity was determined by the payment of a reduced earning capacity pension by the DRV. Based on the actual duration of EBRP-III participation, we defined three separate study groups: 1) no participation, 2) short participation (< 90 days), and 3) long participation (≥ 90 days). A participation duration of 90 days indicated an EBRP-III program participation rate of a minimum of 50%, which was defined as acceptable adherence (long participation).

### Covariates

In our analyses we used a stepwise selection procedure for the selection of covariates. Education level was determined using the available data on the highest level of schooling and/or professional training. Education was categorized as lower secondary or elementary school with or without professional training, higher education entrance qualification with or without professional training, university degree, or no information available.

Subjective medical rehabilitation outcome was determined based on the physician’s assessment indicated in the Phase II rehabilitation discharge letter. Patients could rate their rehabilitation outcome as worse, unchanged, or better.

Information regarding sick leave was based on DRV recordings and expressed as days of absence from work within the last 12 months prior to rehabilitation entry. This data was categorized as no days of absence, less than 3 months of absence, 3–6 months of absence, more than 6 months of absence, or not employed.

The number of comorbidities represented the number of documented physician-based diagnoses next to CHD or ischemic heart disease to represent the degree of multi-morbidity.

### Statistical Analyses

For unadjusted analyses and baseline characteristics, a Chi^2^ test was applied for associations between sex, education, subjective medical rehabilitation outcome (Phase II), sick leave, age at rehabilitation entry, number of comorbidities, and participation in EBRP-III (3 groups). For adjusted analyses, Cox proportional hazards models were applied to account for potential confounding in the relationship between program participation and mortality or reduced earning capacity, respectively [20]. The exposure variable was program participation, with “long participation” as the reference category.

For sensitivity analyses, we used a shorter period of only six months of minimum survival after completing medical rehabilitation (Phase II).

All analyses were performed using the mentioned weighting factor which is provided within the SUF to account for a disproportional sampling procedure [18]. Furthermore, all analyses were performed with the statistical software package SAS University Edition (SAS Institute, Cary, NC, USA). A p-value < 0.05 was considered to indicate statistical significance. For the reporting of patient pathways and outcomes within the German rehabilitation system we used a Sankey diagram. Sankey diagrams are a type of flow diagram in which the width of the arrows is proportional to the flow rate. In our case all CHD patients were divided in two groups (participators vs. non-participators) and the connections (arrows) show the evolution between different stages of the rehabilitation process.

## Results

**Fig. 1:**
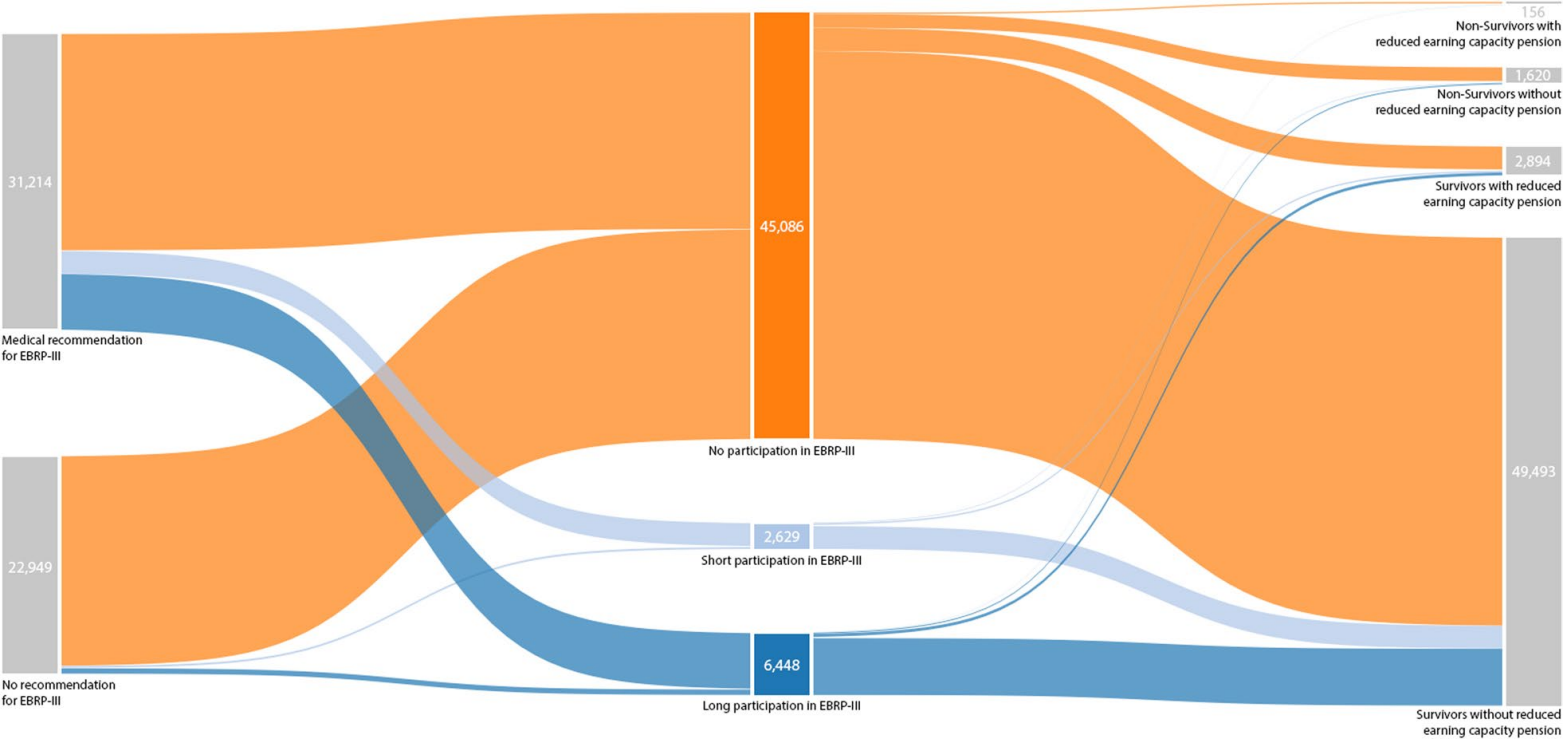
Distribution and adherence to exercise-based cardiac rehabilitation and rehabilitation outcomes of 54,163 CHD patients in Germany, 2010–2017

After the inclusion and exclusion criteria were applied, 54,163 CHD patients remained in the final dataset. Of those, 57.6% received medical recommendations for EBRP-III, 16.8% participated in EBRP-III, and 11.9% participated for more than 90 days (long participation). Furthermore, 1776 (3.3%) patients died, and 3050 (5.5%) received reduced earning capacity pensions during the study period (Fig. 1).

Table 1 shows significant differences in the distribution of demographic characteristics by participation in EBRP-III among CHD patients with the exception for the number of comorbidities. Compared to the average (total), long participation in EBRP-III was more prevalent in women compared to men and older patients were more often adherent to long participation compared to younger patients. Patients with shorter job absence due to sick leave (< 3 months) prior to rehabilitation entry and patients with a better subjective rating of their rehabilitation outcome showed slightly higher long participation rates to exercise-based aftercare compared to patients with longer durations of sick leave or unchanged subjective outcome rating.

**Table 1:** Baseline characteristics (at rehabilitation entry) and group comparison regarding EBRP-III^†^ participation of 54,163 CHD* patients in Germany, 2010–2017

	Total	EBRP-III participation			p-value
		No participation	Short participation (< 90 days)	Long participation (> 90 days)	
N (%)	54,163 (100%)	45,086 (83.2%)	2629 (4.9%)	6448 (11.9%)	
Years under observation (mean ± SD)	4.3 ± 1.8	4.3 ± 1.9	4.4 ± 1.8	4.4 ± 1.8	
*Sex*					< .0001
Men	45,019 (83.1%)	37,785 (83.8%)	2149 (81.7%)	5085 (78.9%)	
Women	9144 (16.9%)	7301 (16.2%)	480 (18.3%)	1363 (21.1%)	
*Age*					< .0001
< 50 years	14,478 (26.7%)	12,353 (27.4%)	796 (30.3%)	1329 (20.6%)	
50–54 years	13,787 (25.5%)	11,548 (25.6%)	716 (27.2%)	1523 (23.6%)	
55–59 years	15,827 (29.2%)	12,951 (28.7%)	744 (28.3%)	2132 (33.1%)	
60–66 years	10,071 (18.6%)	8234 (18.3%)	373 (14.2%)	1464 (22.7%)	
*Comorbidities*					.0904
No comorbidities	2378 (4.4%)	1952 (4.3%)	117 (4.5%)	309 (4.8%)	
1–2 comorbidities	14,386 (26.6%)	11,908 (26.4%)	733 (27.9%)	1745 (27.1%)	
Three or more comorbidities	37,399 (69.0%)	31,226 (69.3%)	1779 (67.7%)	4394 (68.1%)	
*Education*					< .0001
No information	49,564 (91.5%)	40,921 (90.8%)	2520 (95.9%)	6123 (95.0%)	
Lower education	3964 (7.3%)	3606 (8.0%)	94 (3.6%)	264 (4.1%)	
Higher education	493 (0.9%)	451 (1.0%)	7 (0.3%)	35 (0.5%)	
University degree	142 (0.3%)	108 (0.2%)	8 (0.3%)	26 (0.4%)	
*Sick leave prior to rehabilitation entry*					< .0001
Not employed	2059 (3.8%)	1792 (4.0%)	69 (2.6%)	198 (3.1%)	
> 6 months	2899 (5.4%)	2513 (5.6%)	128 (4.9%)	258 (4.0%)	
3–6 months	3477 (6.4%)	2946 (6.5%)	161 (6.1%)	370 (5.7%)	
< 3 months	39,529 (73.0%)	32,502 (72.1%)	2038 (77.5%)	4989 (77.4%)	
No	6199 (11.4%)	5333 (11.8%)	233 (8.9%)	633 (9.8%)	
*Subjective rehabilitation outcome (Phase II)*					.0012
No information	1922 (3.5%)	1578 (3.5%)	99 (3.8%)	245 (3.8%)	
Worse	38 (0.1%)	32 (0.1%)	3 (0.1%)	3 (0.0%)	
Unchanged	7274 (13.4%)	6185 (13.7%)	326 (12.4%)	763 (11.8%)	
Better	44,929 (83.0%)	37,291 (82.7%)	2201 (83.7%)	5437 (84.3%)	

p-value: Chi^2^-Test was used to test for group differences

*CHD: Coronary Heart Disease

^†^EBRP-III: Exercise-based rehabilitation phase III

Table 2 shows the adjusted hazard ratios (HR) for mortality risk as the first primary study outcome. Compared to CHD patients who adhere to EBRP-III for more than 90 days, mortality risk was nearly doubled among CHD patients who did not exercise in structured rehabilitation aftercare programs (HR: 1.97; 95% CI: 1.60–2.43) and 44% higher compared to short participation. Furthermore, men had a 58% higher mortality risk compared to women, and mortality risk increased with age and number of comorbidities. Duration of sick leave prior to rehabilitation entry was also associated with higher mortality risk. For unemployed persons, the mortality risk increased by 68%.

**Table 2:** Association between exercise-based rehabilitation participation (Phase III) and mortality among 54,106 CHD* patients in Germany, 2010–2017

	Cases	HR	95% CI	*p*
*Exercise-based rehabilitation (Phase III)*				
No participation	45,033	**1.97**	**1.60–2.43**	**< .0001**
Short participation (< 90 days)	2627	**1.44**	**1.03–2.01**	**0.034**
Long participation (≥ 90 days)	6446	Ref.		
*Sex*				
Men	44,977	**1.58**	**1.35–1.85**	**< .0001**
Women	9129	Ref.		
*Age*				
Age upon rehabilitation entry (Phase II)	54,106	**1.05**	**1.04–1.06**	**< .0001**
*Comorbidities*				
Number of comorbidities	54,106	**1.07**	**1.02–1.13**	**0.005**
*Education*				
No information	49,514	1.50	0.59–3.79	0.391
Lower education	3957	2.25	0.88–5.72	0.089
Higher education	493	1.35	0.47–3.90	0.584
University degree	142	Ref.		
*Sick leave prior to rehabilitation entry*				
Not employed	2053	**1.68**	**1.31–2.15**	**< .0001**
> 6 months	2897	**1.92**	**1.54–2.39**	**< .0001**
3–6 months	3476	**1.29**	**1.02–1.63**	**0.034**
< 3 months	39,490	0.89	0.76–1.05	0.149
No	6190	Ref.		
*Subjective rehabilitation outcome (Phase II)*				
No information	1918	0.81	0.60–1.10	0.163
Worse	38	2.33	0.68–8.08	0.181
Unchanged	7266	1.10	0.96–1.27	0.167
Better	44,884	Ref.		

*p*-values < .05 indicate statistical significance (bold)

*CHD: Coronary Heart Disease

Table 3 shows the adjusted HR for reduced working capacity as the second primary study outcome. Short participation in EBRP-III was associated with a 24% increase in loss of working capacity compared to long participation. Non-participation in EBRP-III was not associated with a significantly increased risk of loss in working capacity. The risk for loss of working capacity was lower for men compared to women, and this risk decreased by 2% with every additional year of age. Each accompanying disease was associated with an 8% increased risk for loss of working capacity. Duration of sick leave prior to Phase II rehabilitation entry was also associated with higher risk for loss of working capacity.

**Table 3:** Association between exercise-based rehabilitation participation (Phase III) and reduced working capacity among 54,163 CHD* patients in Germany, 2010–2017

	Cases	HR	95% CI	*p*
*Exercise-based rehabilitation (Phase III)*				
No participation	45,086	1.10	0.96–1.26	0.156
Short participation (< 90 days)	2629	**1.24**	**1.00–1.54**	**0.049**
Long participation (≥ 90 days)	6448	Ref.		
*Sex*				
Men	45,019	**0.67**	**0.61–0.74**	**< .0001**
Women	9144	Ref.		
*Age*				
Age upon rehabilitation entry (Phase II)	54,163	**0.98**	**0.97–0.99**	**< .0001**
*Comorbidities*				
Number of comorbidities	54,163	**1.08**	**1.03–1.21**	**0.0003**
*Education*				
No information	49,564	1.20	0.50–2.90	0.680
Lower education	3964	2.02	0.83–4.89	0.120
Higher education	493	0.84	0.30–2.31	0.731
University degree	142	Ref.		
*Sick leave prior to rehabilitation entry*				
Not employed	2059	0.84	0.65–1.09	0.189
> 6 months	2899	**2.57**	**2.15–3.07**	**< .0001**
3–6 months	3477	**2.12**	**1.77–2.53**	**< .0001**
< 3 months	39,529	1.01	0.88–1.16	0.903
No	6199	Ref.		
*Subjective rehabilitation outcome (Phase II)*				
No information	1922	1.05	0.85–1.30	0.658
Worse	38	0.89	0.18–4.43	0.891
Unchanged	7274	1.11	0.99–1.24	0.074
Better	44,929	Ref.		

*p*-values < .05 indicate statistical significance (bold)

*CHD: Coronary Heart Disease

All findings remained similar when we used shorter periods of only 6 months of minimum survival after completing medical rehabilitation (Phase II) for sensitivity analyses.

## Discussion

The focus of the present paper is on the effectiveness of exercise-based cardiac rehabilitation in EBRP-III. Exercise-based cardiac rehabilitation is strongly recommended in national and international guidelines, and its costs are covered by the German pension fund [8, 9]. We used claims data to evaluate the effectiveness of real-world healthcare services. To the best of our knowledge, this is a rarely used study design to address this issue in exercise rehabilitation and the first study of its kind using German data. First of all, our data show an overall low participation rate. Only 16.8% of all CHD patients participated in structured EBRP-III programs and only 11.9% did adhere to these programs for more than 90 days. Second, we see age and sex differences in long-term exercise participation as well as the influence of sick leave prior to rehabilitation entry. Furthermore, our data indicate a beneficial effect of EBRP-III outpatient aftercare programs on CHD patients’ rates of mortality and lost working capacity. This effect was larger for mortality and increased with longer program participation. Patients who did not participate in structured cardiac rehabilitation programs had nearly twice the risk of death of patients who regularly exercised in such programs (HR 1.97, 95% CI 1.60–2.43). A recent study that used a similar approach found similar effects with 68% reduction in mortality risk for patients with atrial fibrillation (AF) and exercise-based cardiac rehabilitation compared to AF patients with no exercise-based cardiac rehabilitation [21].

One potential reason why we do not see this strong effect in our data for working capacity and actually only for short-term EBRP-III participation (HR 1.24, 95% CI 1.00–1.54) may be that mortality is a competing outcome in the non-participating group. Another explanation for that finding could be that CHD is known to be an age-related disease and patients become retired instead of receiving reduced earning capacity pension. Based on our findings and the existing evidence for the effectiveness of physical activity in cardiac prevention and rehabilitation, we conclude that an EBRP-III prescription rate of only 57.6% is not sufficiently high to achieve rehabilitation goals. A previous study already revealed decreasing EBRP-III prescription rates and an overall low participation rate in Germany [16]. Given that guidelines recommend physical activity and (supervised) exercise training for nearly all cardiac patients, EBRP-III prescription appears to be the key component of EBRP-III participation [9]. The vast majority of patients who participated in EBRP-III had received prescriptions, and only a minority had not. It is unclear and undetectable in our data whether all individuals who wanted to become physically active through EBRP-III programs actually had the opportunity to do so. This seems crucial, since representative data for Germany show that only 39% of persons with CHD meet the WHO recommendations for aerobic physical activity [22].

Given that the presence of a medical doctor is compulsory for EBRP-III programs in Germany, more and more EBRP-III providers have had difficulty finding physicians to supervise their outpatient cardiac rehabilitation groups [23]. This is particularly more evident in more rural areas of Germany. A study of Mecklenburg-Western Pomerania showed that the overall number of outpatient rehabilitation sport programs in this area was small, and nearly 50% of the population in this area must travel for more than 60 min to attend one of these programs [24]. The limited availability of EBRP-III programs for cardiac patients led to the development of a position statement by a working group of the German Cardiac Society (DGK), in cooperation with the German Society for Prevention and Rehabilitation of Cardiovascular Diseases (DGPR). Therein, the authors state that physician attendance is not necessary in a so-called “standard cardiac rehab group” and that such groups should be supervised by a qualified non-physician exercise therapist [23]. This regulatory change in program requirements could improve the availability of EBRP-III and thus lead to better rehabilitation outcomes for cardiac patients in Germany.

Another promising approach to improve adherence rates could be the development and implementation of digital tools to make exercise-based cardiac rehabilitation more widely accessible and tailored to individual needs [25]. The European Association of Preventive Cardiology states that mobile technologies like cardiac telerehabilitation can improve cardiac care in rehabilitation phase II and III and lead to reduced mortality and morbidity and improved quality of life. Such approaches are particularly valuable in times of the current COVID-19 pandemic which has led to closure of many cardiac rehabilitation centers [26].

### Strengths and Limitations

The strengths of this study are its large sample size and a relatively unbiased set of secondary claims data. The data concerning the primary study outcomes (death and loss of working capacity) are robust, as they are linked to the official federal death statistic and the payment of a permanent reduced earning capacity pension, respectively. One major limitation of the study is that the dataset comprises only persons whose rehabilitation costs were covered by the German pension fund. This comprises the majority of the working population (usually ≤ 66 years of age), with the exception of civil servants and self-employed persons. Therefore, our results are only generalizable to this particular population. It would be interesting if this also applies to the older (non-working) population. Another limitation of this study is that we have no information regarding the frequency of actual program participation.

## Conclusion

Our data indicate a beneficial effect of regular exercise training in EBRP-III on mortality and working capacity of cardiovascular patients in Germany. Given that overall EBRP-III program participation remains low in this population, our results strengthen the case for making better use of existing exercise-based rehabilitation programs and adapting current program requirements.

## Data Availability

The dataset analyzed in this study was kindly provided upon request by the German pension fund (DRV).

## Abbreviations

CHD: Coronary heart disease
CI: Confidence interval
DALYs: Disability adjusted life years
DGK: German Cardiac Society
DGPR: German Society for Prevention and Rehabilitation of Cardiovascular Diseases
DRV: German pension fund
EBRP-III: Exercise-based rehabilitation phase III
HR: Hazard ratio
ICF: International classification of functioning
SUF: Scientific use file
WHO: World Health Organization
